# Safe Transitions: Optimising Medical Handover in Broadmoor, a High-Secure Forensic Hospital

**DOI:** 10.1192/bjo.2026.11392

**Published:** 2026-06-30

**Authors:** Ahmed Hassan, Isabelle Wood

**Affiliations:** West London NHS Trust, London, United Kingdom

## Abstract

**Aims:**

We aimed to improve the safety, reliability and confidentiality of the medical handover process at Broadmoor, a high-secure forensic hospital. The existing system relied on a mass email sent at the end of on-call shift containing clinical updates, seclusion information and outstanding tasks. We identified key risks: outdated patient lists, missed seclusion reviews, task not reaching the on-call doctor, poor structure and routine sharing of patient full names across a broad distribution list. In a high-secure forensic setting, where patients have complex comorbidities and legal restrictions, communication failures carry heightened clinical and governance risk.

Rather than modifying the email format alone we aimed to redesign the entire handover processto ensure up-to-date information, improve accountability, enhance data security and promote combined verbal and written handover.

**Methods:**

We conducted a Quality Improvement Project using feedback and systemic redesign, we surveyed all regular recipients of the handover email (resident doctors, consultants, site management and physical health nurses). The baseline results showed low satisfaction with the handover email, with 73% reporting dissatisfaction to extreme dissatisfaction, citing safety, accuracy, layout and confidentiality concerns.

Our intervention replaced free-text email handover with a structured process. We introduced an Excel template stored in a secure shared drive which is attached to the email, separate tables were created for acute issues, monitoring needs, seclusion status and tasks. Full patient names were removed and replaced with the initials and hospital numbers. We also introduced a requirement of both verbal and writtenhandover and added a step for the on-call doctor toconfirm seclusion status with site management by the end of their shift. We also reviewed all patient lists for regular monitoring to remove outdated patients. The new process was introduced via hospital-wide presentation.

**Results:**

Post-implementation feedback showed marked improvement, up to 80% of responders reported satisfaction to extreme satisfactionacross domains compared to the baseline dissatisfaction. Staff reported clear structure, more reliable seclusion information, fewer missed tasks and improved confidentiality. The combination of structured documentation and verbal handover reduce reliance on memory during busy shifts and improved multidisciplinary communication. Removal of outdated patients increased clinical relevance of the handover list.

**Conclusion:**

System redesign of the medical handover improved safety, clarity and information governance in a high-secure forensic setting.Structured templates, verbal reinforcement and reduced identifiable patients’ data created a more reliable process.

This low-cost interventiondemonstrated how addressing system factors can strengthen communication, accountability and patient safety across acute and secure psychiatric services.

